# Acute Undifferentiated Febrile Illness in Rural Cambodia: A 3-Year Prospective Observational Study

**DOI:** 10.1371/journal.pone.0095868

**Published:** 2014-04-22

**Authors:** Tara C. Mueller, Sovannaroth Siv, Nimol Khim, Saorin Kim, Erna Fleischmann, Frédéric Ariey, Philippe Buchy, Bertrand Guillard, Iveth J. González, Eva-Maria Christophel, Rashid Abdur, Frank von Sonnenburg, David Bell, Didier Menard

**Affiliations:** 1 Department of Tropical Medicine and Infectious Diseases, University of Munich, Munich, Germany; 2 National Center for Parasitology, Entomology, and Malaria Control, Phnom Penh, Cambodia; 3 Malaria Molecular Epidemiology Unit, Institut Pasteur in Cambodia, Phnom Penh, Cambodia; 4 Parasitology and Mycology Department, Institut Pasteur, Paris, France; 5 Virology Unit, Institut Pasteur in Cambodia, Phnom Penh, Cambodia; 6 Medical Laboratory, Institut Pasteur in Cambodia, Phnom Penh, Cambodia; 7 Foundation for Innovative New Diagnostics (FIND), Geneva, Switzerland; 8 WHO Regional Office for the Western Pacific (WPRO), Manila, Philippines; 9 WHO Country Office, Phnom Penh, Cambodia; 10 Intellectual Ventures Laboratory, Seattle, Washington, United States of America; Centro de Pesquisa Rene Rachou/Fundação Oswaldo Cruz (Fiocruz-Minas), Brazil

## Abstract

In the past decade, malaria control has been successfully implemented in Cambodia, leading to a substantial decrease in reported cases. Wide-spread use of malaria rapid diagnostic tests (RDTs) has revealed a large burden of malaria-negative fever cases, for which no clinical management guidelines exist at peripheral level health facilities. As a first step towards developing such guidelines, a 3-year cross-sectional prospective observational study was designed to investigate the causes of acute malaria-negative febrile illness in Cambodia. From January 2008 to December 2010, 1193 febrile patients and 282 non-febrile individuals were recruited from three health centers in eastern and western Cambodia. Malaria RDTs and routine clinical examination were performed on site by health center staff. Venous samples and nasopharyngeal throat swabs were collected and analysed by molecular diagnostic tests. Blood cultures and blood smears were also taken from all febrile individuals. Molecular testing was applied for malaria parasites, *Leptospira, Rickettsia, O. tsutsugamushi, Dengue*- and *Influenza* virus. At least one pathogen was identified in 73.3% (874/1193) of febrile patient samples. Most frequent pathogens detected were *P. vivax* (33.4%), *P. falciparum* (26.5%), pathogenic *Leptospira* (9.4%), *Influenza* viruses (8.9%), *Dengue* viruses (6.3%), *O. tsutsugamushi* (3.9%), *Rickettsia* (0.2%), and *P. knowlesi* (0.1%). In the control group, a potential pathogen was identified in 40.4%, most commonly malaria parasites and *Leptospira.* Clinic-based diagnosis of malaria RDT-negative cases was poorly predictive for pathogen and appropriate treatment. Additional investigations are needed to understand their impact on clinical disease and epidemiology, and the possible role of therapies such as doxycycline, since many of these pathogens were seen in non-febrile subjects.

## Introduction

Fever is the main clinical symptom of various tropical infectious diseases. In rural areas of developing countries, where diagnostic facilities are limited, etiologies of acute febrile illness remain largely unknown. Malaria has long been considered the most important infectious disease in the Mekong Region in terms of public health impact and it’s elimination has become an international priority with the emergence of artemisinin-resistant parasites along the Thai-Cambodian border [1]. In the past decade, the Cambodian government, with the support of the World Health Organization (WHO) Mekong Malaria Program, successfully implemented diverse strategies to control *falciparum* malaria. Over the last 13 years there was a steady reduction of around 10% per year for diagnosed cases and 8.5% for case fatality rates [2]. By 2010 the overall malaria incidence was 4.07 cases/1,000 population and the mortality rate was 0.98/100,000 population. The relative proportion of *P. vivax* cases rose among reported cases over this time, from 8% in 2000 to 37% in 2011 [3], [4]. The introduction and wide use of malaria rapid diagnostic tests (RDTs), together with the declining overall malaria incidence, has raised the relative importance of non-malarial febrile illness (NMFI) in routine case management. However, the absence of clear guidelines on management of acute undifferentiated fever, and a lack of tools to diagnose non-malarial pathogens to provide information on their prevalence, has resulted in a management dilemma and the suspicion of inappropriate prescription of antimalarial and antimicrobial drugs. A number of infectious diseases such as leptospirosis, scrub typhus, rickettsial diseases, typhoid fever, dengue fever and influenza have been recognized previously among causes of NMFI in the region [5]–[21]. As a first step towards the development of algorithms that could guide clinical management of malaria-negative fever at the peripheral health care level, it is crucial to determine the prevalence and epidemiology of the causative pathogens.

In this context, we designed a cross-sectional prospective observational study to investigate the causes of NMFI in three rural areas of Cambodia. Our primary objective was to detect pathogens in samples taken from febrile outpatients in the remote areas where malaria persists, and where malaria RDTs are the only available diagnostic tool for health workers. We also aimed to determine priorities for diagnostic tools applicable in field conditions and guide the improvement of case management algorithms. Additionally, this study aimed to build capacity for operational research and pathology testing within Cambodia and develop a prototype study design useable in similar settings in other tropical countries.

## Materials and Methods

### Ethics Statement

The study protocol was reviewed and approved by the Cambodian National Ethics Committee on Health Research. An informed written consent was provided by the parents/guardians of all participants before testing. All samples were anonymized prior to laboratory testing.

### Study Sites and Participants

**Figure 1:**
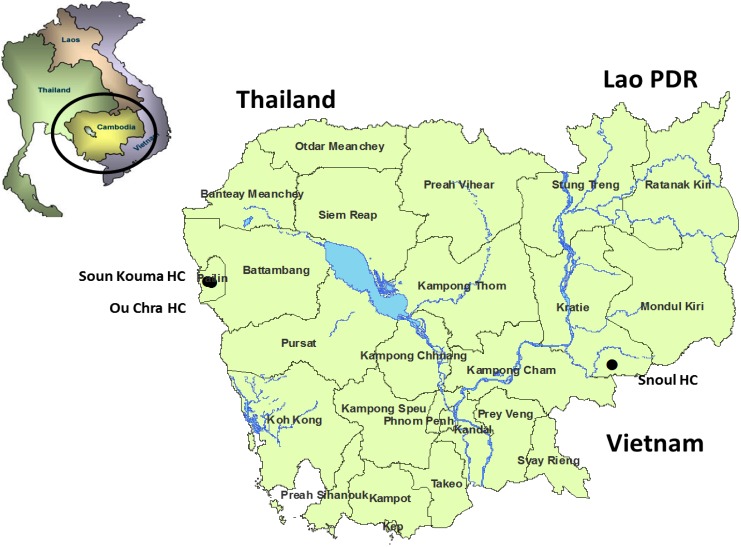
Map of Cambodia displaying the location of the study sites in remote areas in western (Soun Kouma and Ou Chra) and eastern Cambodia (Snoul).

Three outpatient health centers (HCs) in remote areas in western (Soun Kouma and Ou Chra, Pailin Province) and eastern Cambodia (Snoul, Kratie Province) were selected as study sites (Figure 1). These sites were chosen to demonstrate potential geographical differences in pathogen distribution (east and west). Pailin Province is known to be an area of low malaria transmission, whereas Kratie province is an area of intermediate transmission. Further considerations for choice of sites were availability of staff and sample transportation service which were coordinated by the National Malaria Control Program (CNM).

From January 2008 to December 2010, all male and female patients, aged from 7 to 49 years, who had an acute febrile illness (tympanic membrane temperature on admission >38.0°C, and a history of fever not longer than 8 days), and who were eligible to be tested for malaria by RDT according to the national guidelines (CareStart Malaria HRP2/pLDH (Pf/PAN) Combo G0131, Access Bio Inc. USA) were invited to participate in the study. Patients with severe illness warranting immediate transfer to a hospital were excluded.

In parallel, a group of non-febrile accompanying persons, aged 7–49 years, with a tympanic membrane temperature <38.0°C, no recent history of febrile illness, and providing written informed consent, were recruited as controls.

### Field Procedures

In both groups (cases and controls groups) a physical examination and malaria RDT (CareStart Malaria HRP2/pLDH (Pf/PAN) COMBO G0131, Access Bio Inc. USA) were performed as per routine practice by the clinical staff. History of illness and results of the physical examination (for case group) were recorded on standardized and simplified forms in Cambodian language, provided by the National Malaria Control Programme (Figure S1). Whole blood (10–15 ml), a blood smear and a nasopharyngeal throat swab were collected from each study participant. Blood samples were separated immediately, with 5 ml of whole blood collected in an EDTA tube, stored at 4°C, and transported to the Institut Pasteur in Cambodia (IPC) in Phnom Penh within 24 hours. Another 5 ml of blood was centrifuged on site and the plasma stored in liquid nitrogen, as were the throat swab samples before shipment to IPC. An additional 5 ml of whole blood from febrile patients was immediately incubated in an aerobic blood culture bottle (Pharmaceutical Factory No. 2, Vientiane, Lao PDR), sent to Phnom Penh in an insulated box (with an electronic temperature monitor), and incubated and monitored at 37°C, for 5 days. The patient’s data and the specimens were anonymized on site. Clinical data forms were sent to CNM where translation and data entry were performed.

### Laboratory Procedures

At the IPC in Phnom Penh, EDTA blood samples were centrifuged at 2000 rpm for 10 minutes and DNA extraction was performed on 200 µl of packed blood cells, using the QIamp DNA Mini Kit (Qiagen, Hilden, Germany), according to the manufacturer’s instructions. Nested-PCRs and nucleotide sequencing were applied to detect DNA sequences of *Plasmodium* (*cytb* gene [22]), *Leptospira* (*16srRNA* gene [23]), *O. tsutsugamushi* (*47 kDa* gene [24]) and *Rickettsia* (*gltA* and *ompB* genes [24]). After purification by filtration with a NucleoFast 96 PCR plate (Macherey-Nagel, Düren, Germany), sequencing reactions were performed for both strands by using the ABI Prism BigDye terminator cycle sequencing ready reaction kit run on a 3730 xl genetic analyzer (Applied Biosystems, Courtaboeuf, France). Electrophoregrams were visualized and analyzed with CEQ2000 genetic analysis system software (Beckman Coulter, Villepinte, France). Nucleotide sequences were compared to the *P. falciparum* 3D7 sequence (GenBank accession number AY282930), *Leptospira interrogans* 16sRNA sequence (GenBank accession number EU581713), *Orientia tsutsugamushi* 47 kDa sequence (GenBank accession number L11697) and *Rickettsia* sp. OmpB and GltA sequences (GenBank accession numbers EF219461 and AB444098). Plasma and throat swab samples were processed for RNA extraction using the QIamp Viral RNA Mini Kit (Qiagen, Hilden, Germany) on 200 µl of plasma and from 140 µl of throat swab-sample, according to the manufacturer’s instructions. In-house multiplex Reverse-Transcriptase (RT) PCR assays were performed on plasma and throat swab RNA extracts, for detection of Dengue (*PrM/E* gene [25]) and Influenza-Virus (*M* gene [26]), respectively [27]. In case of detection of Influenza A virus, sub-typing was performed by real-time-PCR [27]. Positive and negative samples were used as controls in each run.

The thresholds of detection for each PCR/RT-PCR were assessed by using dilutions of positive controls (Table S1). External quality control assurance system was also set up between Phnom Penh and Vientiane (Lao-Oxford-Mahosot Hospital-Wellcome Trust Research Unit - LOMWRU, Microbiology Laboratory, Mahosot Hospital, Vientiane, Lao PDR). C-reactive-protein (CRP) was also quantified on plasma using latex bead immunoturbidimetric method adapted on Integra 400 (Ref 0764930, Roche Diagnostics, Indianapolis, Ind., USA) and calibrated controls, according to the manufacturer instructions. Finally, blood smears were analyzed by a single WHO Level 1 qualified microscopist for comparative malaria diagnostics.

### Patient Management

Available laboratory results which may have had a direct bearing on patient management were immediately sent back to the HCs. Immediate decisions on the use of antimalarial or antimicrobial drugs were taken by qualified personnel at the HCs as per usual practice.

### Statistical Analysis

Data were entered and verified using Microsoft Excel software and analyzed using XLSTAT (Addinsoft, France) and MedCalc (MedCalc 9.1.0.1, Belgium). The Mann-Whitney *U* test or Kruskal-Wallis method were used for non-parametric comparisons, and Student’s t test or one-way analysis of variance for parametric comparisons. For categorical variables, Chi-squared or Fisher’s exact tests were used to assess significant differences in proportions. Odd ratios (OR) and their 95% confidence intervals (CI) were measured to compare prevalence of pathogens between case and control groups. The Attributable Fraction among case (AFe) was assessed for each pathogen using the following formula: [(OR-1)/OR]×100. Hypothesis testing was made using risk differences, exact 95% confidence intervals, and p-values. A p*-*value (two-tailed) of less than 0.05 was considered statistically significant.

## Results

### Study Population

From January 2008 to December 2010, 1475 individuals were recruited. Among them, 1193 (80.8%) were febrile patients (case group): 621 in Soun Kouma HC and 650 in Ou Chra HC in Pailin Province and 204 in Snoul HC in Kratie Province, and 282 (19.1%) non-febrile individuals (control group) (Figure S2). As presented in Table 1, significant differences in patient characteristics were found between sites: (i) mean temperature on admission was higher in Soun Kouma HC (p<0.0001), (ii) time delay in days between the onset of the fever and the consultation was higher in Snoul HC (p<0.0001), (iii) sex ratio (M/F) was higher in Snoul HC (p<0.0001) and (iv) mean age of febrile patients was lower in Soun Kouma HC (p<0.0001). The frequency of clinical symptoms of febrile patients on admission also differed between sites (p<0.0001). The most common symptoms observed were sore throat (612/1193, 51.3%), cough (585/1193, 49.0%), and running nose (259/1193, 21.7%).

**Table 1:** Characteristics of patients and controls in each study sites.

Characteristic and on-site management		Soun Kouma HC		Ou Chra HC		Snoul HC		Total		p*-value* *
		Cases	Controls	Cases	Controls	Cases	Controls	Cases	Controls	
Number		450	171	576	74	167	37	1193	282	n/a
Temperature	Mean (SD), °C	39.0 (0.5)	-	38.8 (0.5)	-	38.8 (0.5)	-	38.9 (05)	-	<0.0001
Duration of fever	Mean (SD), days	2.4 (1.0)	-	2.7 (1.0)	-	3.0 (1.6)	-	2.6 (1.1)	-	<0.0001
Sex	M	254	63	404	8	143	34	801	105	<0.001
	F	196	108	172	66	24	3	392	177	
	Ratio (M/F)	1.3	0.6	2.3	0.1	6.0	11.0	2.0	0.6	
Age	Mean (SD) years	21.1 (10.7)	34.5 (11.6)	24.6 (10.6)	25.7 (7.5)	25.3 (9.5)	28.1 (10.0)	23.4 (10.6)	31.4 (11.2)	<0.001
Clinical symptoms	Fever (>37,5°C)	100	-	100	-	100	-	100	-	<0.0001
	Sore throat (%)	67.8	-	46.4	-	24.0	-	51.3	-	
	Cough (%)	59.1	-	46.4	-	31.1	-	49.0	-	
	Running nose (%)	42.0	-	7.3	-	16.8	-	21.7	-	
	Diarrhoea (%)	17.6	-	22.2	-	10.8	-	18.9	-	
	Vomiting (%)	25.3	-	16.1	-	3.6	-	17.9	-	
	Ear ache	13.6	-	0.2	-	2.4	-	5.5	-	
	Headache (%)	6.4	-	0.5	-	1.8	-	2.9	-	
Clinical diagnosis on-site	Malaria (%)	23.6	-	37.5	-	65.9	-	36.2	-	<0.0001
	Enteric fever (%)	1.4	-	18.9	-	13.2	-	11.5	-	
	UARI (%)	74.4	-	43.6	-	19.7	-	51.9	-	
	LARI (%)	0.6	-	0.0	-	1.2	-	0.4	-	
Treatment on-site	Antifebrile drugs (%)	100	-	100	-	98.8	-	99.8	-	<0.0001
	Antimalarial drugs (%)	23.2	-	37.7	-	66.5	-	36.1	-	
	Antimicrobial drugs (%)	75.9	-	62.7	-	34.1	-	63.7	-	
	No drug (%)	0.9	-	0	-	0	-	0.34	-	

n/a: Not calculated;

*among cases between sites.

### Clinical Diagnosis and Management of Febrile Diseases

**Figure 2:**
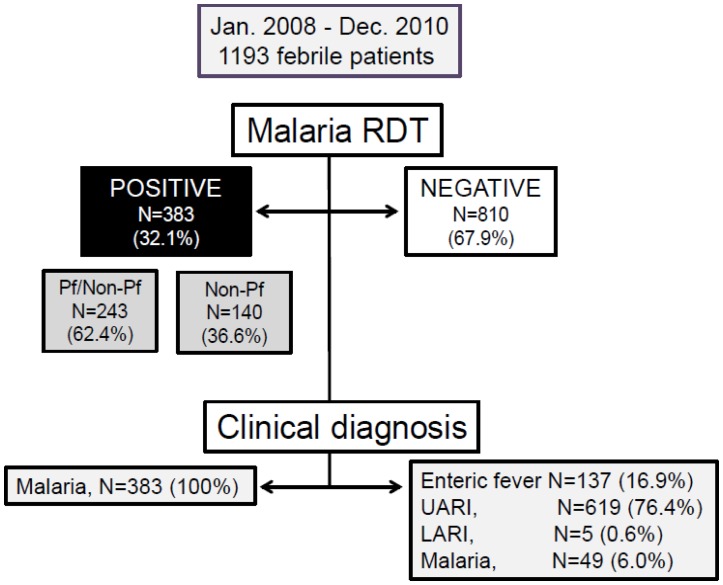
Diagnoses for febrile patients in the study sites, with use of malaria RDT and clinical symptoms.

Of febrile cases, 383/1193 (32.1%) had a positive malaria-RDT on admission (Figure 2) and were treated according to national guidelines [28]. Febrile individuals that had a negative malaria-RDT were clinically diagnosed by health centre staff using normal procedure as: (i) acute upper respiratory infections (AURI) (619/810, 76.4%), (ii) enteric fever (137/810, 16.9%), (iii) malaria (49/810, 6.0%) and (iv) acute lower respiratory infections (ALRI) (5/810, 0.6%). Patients in the AURI group were usually treated with the following antimicrobial drugs: amoxicillin (AMX) (478/619, 77.2%), sulfamethoxazol/trimethoprim (SMX) (89/619, 14.4%), penicillin (30/619, 4.8%), erythromycin (8/619, 1.3%) and cloxacillin (6/619, 1.0%). Metronidazol was used in 3 cases and chloroquine (CQ) in 2 cases. The remaining 3 patients received paracetamol only. Patients clinically diagnosed with enteric fever were treated with metronidazol (60/137, 43.8%), SMX (48/137, 35.0%), fluoroquinolones (21/137, 15.3%), AMX (4/137, 3.0%), nalidixic acid (1/137, 0.7%), erythromycin (1/137, 0.7%), CQ (1/137, 0.7%) or mebendazol (1/137, 0.7%). Patients clinically diagnosed as malaria, despite negative RDT (49/810) were treated with antimalarials according to the national guidelines. All patients with lower acute respiratory infections (5/810) were treated with AMX.

### Detection of Potential Pathogens

In total, 754/1475 (51.1%) enrolled individuals were malaria positive by PCR. The observed prevalence was almost 2-fold higher in the case group (676/1193, 56.7%) compared to the control group (78/282, 27.7%, p<0.01). The attributable fraction among cases (AFe) was estimated to 70% (Table 2). Nucleotide sequencing revealed that the predominant species was *P. vivax* (389/754, 51.6%) followed by *P. falciparum* (306/754, 40.6%). Both *Plasmodium spp.* were more frequently detected in the case than in the control group (p<0.01), with AFe values of 72% and 61%, respectively. In 7.4% of all malaria positive samples (56/754), *P. falciparum/P. vivax* mixed infections were detected without difference in term of frequency between the groups (p = 0.07). Two human cases of the emerging simian malaria parasite *P. knowlesi* were also detected, one in the case and one in the control group. *P. ovale* was found only once, in the control group (Table 2).

**Table 2:** Prevalences of the detected pathogens in the cases and controls study population and attributable fractions among cases, Cambodia, January 2008–December 2010.

Detected pathogen	Total		Cases (n = 1,193)		Controls (n = 282)		*p*-value	OR (95% CI)	*p*-value	Attributable fraction among cases (AFe)
	(N = 1,475)									
	N	%	N	%	N	%				
***Plasmodium*** ** spp.**	**754**	**51.1**	**676**	**56.7**	**78**	**27.7**	**<0.01**	**3.4 (2.6–4.5)**	**<0.01**	**70%**
*P. vivax*	389	26.4	359	30.1	30	10.6	<0.01	3.6 (2.4–5.4)	<0.01	72%
*P. falciparum*	306	20.7	277	23.2	29	10.3	<0.01	2.6 (1.7–4.0)	<0.01	61%
*P. vivax + falciparum*	56	3.8	39	3.3	17	6.0	0.07	n/a	n/a	n/a
*P. ovale*	1	0.1	0	0.0	1	0.4	n/a	n/a	n/a	n/a
*P. knowlesi*	2	0.1	1	0.1	1	0.4	n/a	n/a	n/a	n/a
**Pathogenic ** ***Leptospira*** ** spp.**	**140**	**9.5**	**112**	**9.4**	**28**	**9.9**	**0.42**	**n/a**	**n/a**	**n/a**
*L. interrogans*	85	5.8	61	5.1	24	8.5	0.20	n/a	n/a	n/a
*L. weilii*	47	3.2	44	3.7	3	1.1	0.08	n/a	n/a	n/a
*L. kmetyi*	3	0.2	2	0.2	1	0.4	n/a	n/a	n/a	n/a
*L. kirschnerii*	1	0.1	1	0.1	0	0.0	n/a	n/a	n/a	n/a
*L.santosaraii*	1	0.1	1	0.1	0	0.0	n/a	n/a	n/a	n/a
*L. genomospecies1*	1	0.1	1	0.1	0	0.0	n/a	n/a	n/a	n/a
*L. wolffii*	1	0.1	1	0.1	0	0.0	n/a	n/a	n/a	n/a
*L. noguchii*	1	0.1	1	0.1	0	0.0	n/a	n/a	n/a	n/a
***O*** **. ** ***tsutsugamushi***	**54**	**3.7**	**47**	**3.9**	**7**	**2.5**	**0.48**	**n/a**	**n/a**	**n/a**
***Rickettsia*** ** spp.**	**3**	**0.2**	**2**	**0.2**	**1**	**0.4**	**n/a**	**n/a**	**n/a**	**n/a**
**Dengue virus 1–4**	**80**	**5.4**	**75**	**6.3**	**5**	**1.8**	**0.01**	**3.7 (1.5–9.3)**	**0.005**	**73%**
**Influenza A virus**	**87**	**5.9**	**83**	**7.0**	**4**	**1.4**	**0.01**	**5.2 (1.9–14.4)**	**0.001**	**81%**
**Influenza B virus**	**26**	**1.8**	**23**	**1.9**	**3**	**1.1**	**0.77**	**n/a**	**n/a**	**n/a**
**Cultured bacteria**	**9**	**0.8**	**9**	**0.8**	**0**	**-**	**n/a**	**n/a**	**n/a**	**n/a**

n/a: Not calculated.

All positive malaria RDT individuals (383/1475, 25.9%) belonged to the case group (383/1193, 32.1%) and 376/383 (98.2%) of them were confirmed positive by malaria-PCR. In addition, 300 patients from the case group with negative malaria RDT results were also positive by malaria PCR.

*Leptospira* spp. were identified as the second most frequent pathogens. Globally, 167/1475 samples were positive by PCR (11.3%). The ratio between pathogenic and non-pathogenic *Leptospira* was 140/27. The distribution of the main pathogenic species determined by sequencing was as following: *L. interrogans* (85, 60.8%), *L. weilii* (47, 33.6%), *L. kmetyi* (3, 2.1%), *L. kirschneri* (1, 0.7%), *L. santarosai* (1, 0.7%), *L. wolffii* (1, 0.7%), *L. genomospecies1* (1, 0.7%) and *L. noguchii* (1, 0.7%) (Table 2). The overall prevalence of pathogenic leptospirosis was not significantly different between the three sites, but there was an uneven distribution between species (p<0.01): *L. interrogans* was the most prevalent species in Snoul (22/23) compared to Pailin (29/74). Interestingly, the prevalence of *L. interrogans* was higher in the control group (24/282, 8.5%) than in the case group (61/1193, 5.1%) (OR = 0.60, 95% CI: 0.37–0.98, p = 0.04), while the prevalence of other pathogenic *Leptospira* spp. was higher in cases than controls (4.3% *vs.* 1.4%, OR = 2.5, 95% CI: 1.1–5.6, p = 0.02). The AFe were not evaluated.

*O. tsutsugamushi* was detected in 54/1475 samples (3.7%). Its prevalence was not associated with site or with fever. Moreover, 3 samples were positive for other *Rickettsia* species.

Virological tests results were available for 1470 plasma and 1473 throat swab samples. Of the plasma samples, 80/1470 (5.4%) were found positive for Dengue virus type 2 (44/80, 55.0%), type 1 (22/80, 27.5%), type 4 (8/80, 10.0%) and type 3 (6/80, 7.5%). The overall prevalence was not significantly different between sites, but more frequent in febrile patients (75/1190, 6.3%) than in non-febrile individuals (5/280, 1.8%, p = 0.01, OR = 3.7, 95% CI: 1.5–9.3, AFe = 73%). The prevalence of type 2 and type 4 was highest in Ou Chra and Soun Komar HC in Pailin province, respectively. The temporal distribution of the dengue cases clearly showed an annual epidemic peaking between May and October (Figure S3).

Both Influenza A (87/1473, 5.9%) and B serotypes (26/1473, 1.8%) were detected in the throat swabs. The overall prevalence was significantly different between sites: 11.3% in Ou Chra HC, 5.5% in Soun Komar HC and 3.4% in Snoul HC (p<0.01) and between groups (106/1191, 8.9% case group *vs.* 7/282, 2.5% in control group, p = 0.001). Influenza A cases were more frequent in febrile patients (83/1193, 7.0%) than in non-febrile individuals (4/280, 1.4%, p = 0.01, OR = 5.2, 95% CI: 1.9–14.4, AFe = 81%). As observed with Dengue, the temporal distribution of Influenza cases clearly showed a seasonal emergence between September and November (Figure S4).

Among 1128 blood cultures collected from febrile patients, only 9/1128 samples (0.8%) containing pathogenic bacteria were considered as evidence of community acquired septicemia (CAS): *S. pneumoniae* (n = 1), *E. coli* (n = 1), *E. cloacae* (n = 5), *S. typhi* (n = 1) and *S. paratyphi* (n = 1). In 2 cases (*E. cloacae* and *S. paratyphi*) was associated with *P. vivax* infection. Other positive bacteria growths (98/1128) identifying as *Staphylococcus non-aureus, Corynebacteria* spp., *Pseudomonas fluorescens* and *Pseudomonas putida* were considered as contamination. These results were immediately reported back to the health center and all patients were provided the appropriate antimicrobial treatment.

The distribution and the proportion of pathogens found in samples from febrile patients enrolled in 3 sites are presented in Table 3.

**Table 3:** The distribution and the proportion of pathogens found in samples from febrile patients enrolled in 3 sites, Cambodia 2008–2010.

Pathogens		Sampling sites			Total	
		Soun Kouma HC	Ou Chra HC	Snoul HC		
		N = 576	N = 450	N = 167	N = 1193	
no pathogen		148	146	25	319	26.7%
one pathogen	*Plasmodium sp.*	280	156	107	543	45.5%
	*Influenza viruses*	15	48	3	66	5.5%
	*Leptospira*	26	11	7	44	3.7%
	*Dengue viruses*	17	23	2	42	3.5%
	*Orientia tsutsugamushi*	12	12	1	25	2.1%
	*Enterobacter cloacae*	2	0	0	2	0.2%
	*Escherichia coli*	0	1	0	1	0.1%
	*Pseudomonas fluorescens*	1	0	0	1	0.1%
	*Pseudomonas putida*	1	0	0	1	0.1%
	*Rickettsia*	1	0	0	1	0.1%
	*Streptococcus pneumoniae*	0	1	0	1	0.1%
	*Salmonella typhi*	0	1	0	1	0.1%
two pathogens	*Plasmodium sp. – Leptospira*	29	15	8	52	4.4%
	*Plasmodium sp. - Influenza viruses*	16	10	3	29	2.4%
	*Plasmodium sp. - Dengue viruses*	14	8	6	28	2.3%
	*Plasmodium sp. - O. tsutsugamushi*	7	6	1	14	1.2%
	*Plasmodium sp. - E. cloacae*	1	0	0	1	0.1%
	*Plasmodium sp. - S. paratyphi*	0	0	1	1	0.1%
	*Dengue viruses – Leptospira*	0	1	1	2	0.2%
	*Dengue viruses - Influenza viruses*	0	1	0	1	0.1%
	*E. cloacae - Influenza viruses*	1	0	0	1	0.1%
	*O. tsutsugamushi – Leptospira*	0	1	1	2	0.2%
	*O. tsutsugamushi - E. cloacae*	1	0	0	1	0.1%
	*Rickettsiae - Influenza viruses*	0	1	0	1	0.1%
	*Influenza viruses – Leptospira*	1	4	0	5	0.4%
three pathogens	*Plasmodium sp. - O. tsutsugamushi – Leptospira*	2	1	0	3	0.3%
	*Plasmodium sp. - Dengue viruses – Leptospira*	0	0	1	1	0.1%
	*Plasmodium sp. - Influenza viruses – Leptospira*	0	2	0	2	0.2%
	*Plasmodium sp. - O. tsutsugamushi - Dengue viruses*	0	1	0	1	0.1%
	*Plasmodium sp. - O. tsutsugamushi - Influenza viruses*	1	0	0	1	0.1%

### Co-infections

A substantial proportion of co-infections (more than one pathogen detected by PCR) was observed in this study. *O. tsutsugamushi* was present as the only pathogen in 31 samples and in co-infection with other pathogens in 28 cases (n = 19, *P. falciparum* and *P. vivax;* n = 7, Leptospira; n = 1, Dengue virus and n = 1, *E. cloaceae*). Dengue virus was also frequently observed in co-infections (32/56, 57%), especially with malaria parasites (n = 27, *P. falciparum* and *P. vivax*). Similarly, Influenza virus was also frequently detected in co-infections (42/114, 37%), especially with malaria parasites (n = 32, *P. falciparum* and *P. vivax*). Other co-infections included *Leptospira* (n = 6), *O. tsutsugamushi* (n = 1), Dengue virus (n = 1), *E. cloacae* (n = 1) and *Rickettsia* (n = 1).

### C-Reactive Protein (CRP) Quantitative Detection

Quantitative detection of CRP was performed on 1475 plasma samples. The results are presented in Table 4. Significant differences in median were observed between febrile patients and non-febrile individuals (25.7 mg/l *vs.* 1.4 mg/l, p<0.01). The lowest median CRP concentrations were observed in viral infections (Dengue = 8.2 mg/l and Influenza = 9.6 mg/l) being significantly different to CRP levels in patients with malaria (37.9 mg/l p<0.01) and scrub typhus (26.4 p<0.006). Median CRP concentration was intermediate in febrile patients where no pathogen was detected (6.3 mg/l) or with multiple infections (16.2 mg/l). The sensitivity, specificity, positive predictive (PPV) and negative predictive values (NPV) of CRP>5 mg/ml for bacterial mono-infection versus viral infections were 87.8% (CI95%: 78.2–94.3%), 30.8% (CI95%: 22.3–40.5%), 46.8% (CI95%: 38.3–55.4%) and 78.6% (CI95%: 63.2–89.7%), respectively. The interactive dot diagram analysis indicating the cut-off point with the best separation (minimal false negative and false positive results) was set up at 21.3 mg/L with 52.5% of sensitivity and 84.3% of specificity.

**Table 4:** CRP levels in different patient subgroups.

Patient subgroup	N	Median CRP concentration	IQR	Range
Global	1475	17.7 mg/L	3.3–53.3 mg/L	0.1–389 mg/L
Febrile patients	1193	25.7 mg/L	7.6–69.5 mg/L	0.2–389 mg/L
Non-febrile individuals	282	1.4 mg/L	1.0–5.0 mg/L	0.2–236 mg/L
Malaria	612	37.9 mg/L	8.9–86.3 mg/L	0.3–366 mg/L
Dengue virus	44	8.2 mg/L	3.0–15.5 mg/L	1.0–75.5 mg/L
Influenza virus	71	9.6 mg/L	3.6–15.8 mg/L	0.2–68.1 mg/L
Leptospirosis	63	19.2 mg/L,	1.4–62.8 mg/L	0.5–284 mg/L
Scrub typhus	31	26.4 mg/L	6.3–78.9 mg/L	1.0–244 mg/L
Febrile patients where no pathogen was detected	487	6.3 mg/L	1.4–28.6 mg/L	0.1–376 mg/L
Multiple infections	167	16.2 mg/L	5.3–44.7 mg/L	0.2–389 mg/L

## Discussion

The diagnosis of non-malaria febrile illness in resource-limited rural areas remains challenging [29]. One of the main findings of this study was the observation that whilst the clinical management of malaria is reportedly working well in Cambodia, the majority of malaria-RDT negative febrile patients did not receive an appropriate diagnosis and treatment. Clinical diagnosis together, with the RDTs performed in the field, succeeded in identifying and appropriately treating 56.6% (383/676) of the malaria cases. However, even when RDTs are currently the most important tool available in peripheral health centers to diagnose malaria especially caused by *P. falciparum* (243/316, 76.9%), an important proportion of low-density malaria infections is still missed as demonstrated by PCR in this study. Most of the RDT-negative febrile cases were carrying malaria parasites that were detected by PCR but not by RDT (314/810, 38.7%, mainly *P. vivax*; 188/314, 60%). Although it is not clear if those low-density malaria infections are actually the cause of fever in those patients (AFe among malaria case 70%), these results emphasize the need for more sensitive diagnostic tools for detection and treatment of all infections to target elimination. We observed that most of the RDT-negative cases were clinically diagnosed as UARI and treated apparently indiscriminately with antimicrobial drugs (mainly AMX). Only 14% of them received an effective treatment against the pathogen identified by PCR. These findings underline the need of specific diagnostic tools and clinical training of the health center staff for the effective management of febrile patients and rational use of antimicrobials. Leptospirosis was frequently observed in people with AUFI, regardless the clinical status (case and control groups). This could be due to the recruitment of controls among family members or other accompanying persons, who had been exposed to the same risk-factors as the patients. The high prevalence of leptospirosis detected in this study was consistent with previous reports from similar settings in Thailand, Lao PDR [9], [10], [12], and Cambodia [5]. This underlines the importance of this disease in the region. However, the high frequency observed in the asymptomatic/non-febrile group is new. The awareness about leptospirosis needs to be raised and further research on its epidemiology, clinical symptoms and outcomes in Cambodia, including its significance in afebrile people, should be undertaken. Furthermore, suitable diagnostic tools for leptospirosis should be made available in peripheral health services. Since no serological tests were applied, no information on the serovar could be obtained.

A high frequency of dengue fever was observed in our sampled population, mostly adult and rural, while dengue is known to be more common in urban settings and young children [11]. These results underline the importance of awareness by health center staff to be able to distinguish viral infections and be aware of their possible complications. Dengue and influenza infections had annual epidemic waves, implying that at the corresponding time of the year, HC staff should consider these viral diseases as important differential diagnoses for malaria. Both viral infections were also associated with a significantly lower CRP-level compared to malaria or bacterial infections. CRP could be a helpful tool to distinguish viral infections as cause of NMFI in the future however further studies to define more accurate thresholds are required.

Co-infections were also frequent in this study (∼14%). Most frequently, these included malaria parasites (*P. falciparum* and *P. vivax*) and a second non-malarial pathogen such as *Leptospira* spp., *Rickettsia* spp., *O. tsutsugamushi* or dengue virus. Reports of malaria co-infections with other pathogens such as HIV or helminthes are numerous especially from sub-Saharan Africa. In Southeast Asia, however, reports of co-infections in malaria patients remain rare. Malaria in Southeast Asia is often associated with outdoor occupations including logging, mining and agriculture, activities that also expose people at risk for leptospirosis, rickettsiosis and scrub typhus. In many cases of malaria co-infections, *P. vivax* was the most frequent species found by PCR (very at low density of infection). These cases could be interpreted as chronic asymptomatic infections with *P. vivax* plus a simultaneous acute infection with another pathogen. Unfortunately nothing is known about the interactions of these pathogens in co-infections so far, but it can be assumed that multiple infections complicate malaria and may lead to failure to respond to treatment [30]. The awareness of the possibility of co-infections should be raised and physicians should be suspicious of it in malaria cases with poor treatment response or atypical manifestations. Additional tests to improve guidance for antimicrobial therapy are needed. In their absence, empirical treatment with doxycycline could be a valid therapeutic option [31].

Although a broad test battery was applied for the detection of pathogens causing fever, it was not possible to detect a causative agent in ∼27% of febrile patients. A variety of other pathogens come into consideration as cause of fever, not all of which were evaluated here. For example, HIV infections, viral hepatitis and tuberculosis were not evaluated due to ethical approval conditions but are likely to contribute to the overall burden of NMFI. Japanese Encephalitis Virus (JEV) was also a possible cause, especially in cases of prolonged fever or additional signs of meningitis. However convalescent serum samples were not available for additional testing. A combined *Flavivirus* MAC-ELISA (JE–Dengue IgM Combo ELISA; Panbio Diagnostics, Australia) was run on 10 samples of patients with a history of more than 5 days of fever and 2 had a positive result for JEV and 3 samples were positive for dengue virus. Another viral disease of interest to evaluate in the future is Chikungunya virus infection, of which several outbreaks were reported from Thailand in 2008 and 2009 [32]. Further possible causes of NMFI include viral infections by Epstein-Barr-virus, Hepatitis A, Hepatitis E, and Coxsackie virus, and other bacterial infections such as Q-fever, brucellosis and melioidosis.

The clinical evaluation of the subjects recruited for this study has not revealed any predictor symptoms or specific risk factors to differentiate between the different causes of NMFI. The evaluation of CRP-levels, as the only biological marker for infection in this study, did show an informative value for the presence of any infection (versus no pathogen identified), as well as for the cause of the infection (viral *vs.* bacterial). However, this correlation is still weak to be used to assign a definite diagnosis and guide treatment decisions. Accessory information such as a full blood cell count and other biomarkers could provide additional useful diagnostic clues that may help to refine decision-making.

This first NMFI study conducted in Cambodia provides highly valuable information for the planning and design of further investigations. Further studies where district hospitals and HCs are compared for disease burden and diagnosis of severe infections should also be considered. Seasonality of fever cases as demonstrated in this study should also be considered to target resources and increase cost-effectiveness. Paired serum samples should be collected from all patients to define previous or recent exposure to pathogens and guide epidemiological studies. Based on the results of this study, future studies should also collect data on: (i) regional disease burden and seasonality of infectious diseases, (ii) clinical condition of patients and severity of disease (case fatality rates with and without treatment), and (iii) availability and level of health care facilities and services. Studies should assess disease burden and laboratory gaps across each level of health care. To develop effective clinical guidelines it is imperative to involve physicians, nurses and other health care professionals from various levels of health care as well as health policy makers [29]. Intervention packages have to be in line with the current clinical management efforts and the Ministry of Health’s planning for human resources, facility upgrades and laboratory strengthening.

## Supporting Information

Figure S1 **NMFI clinical standardized form.**
(TIFF)

Figure S2 **Flow chart of cases and controls enrolled in the study sites (January 2008–December 2010).**
(TIF)

Figure S3 **Temporal distribution of the dengue cases observed in the study sites (January 2008–December 2010).**
(TIF)

Figure S4 **Temporal distribution of the influenza (A and B) cases observed in the study sites (January 2008–December 2010).**
(TIF)

Table S1 **Estimated detection thresholds of nucleic acid amplication tests (NAAT) used in the study.**
(DOCX)
