# Supplementary figures and images for: Acute Undifferentiated Febrile Illness in Rural Cambodia: A 3-Year Prospective Observational Study
Source: PLoS One. 2014 Apr 22;9(4):e95868. doi: 10.1371/journal.pone.0095868 (PMC3995936; doi:10.1371/journal.pone.0095868)

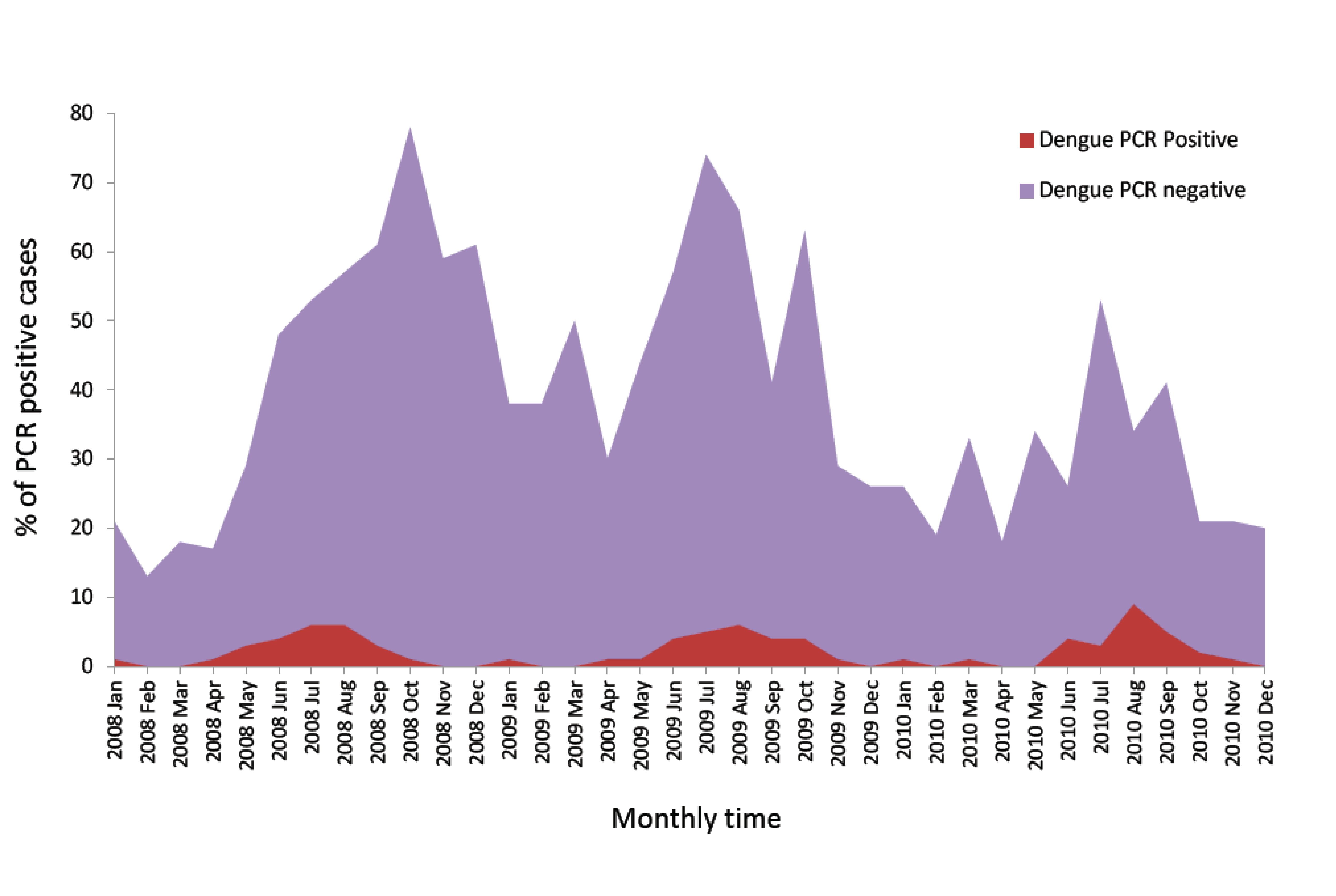

Supplement: Figure S3 — Temporal distribution of the dengue cases observed in the study sites (January 2008–December 2010). (TIF) [file pone.0095868.s003.tif]

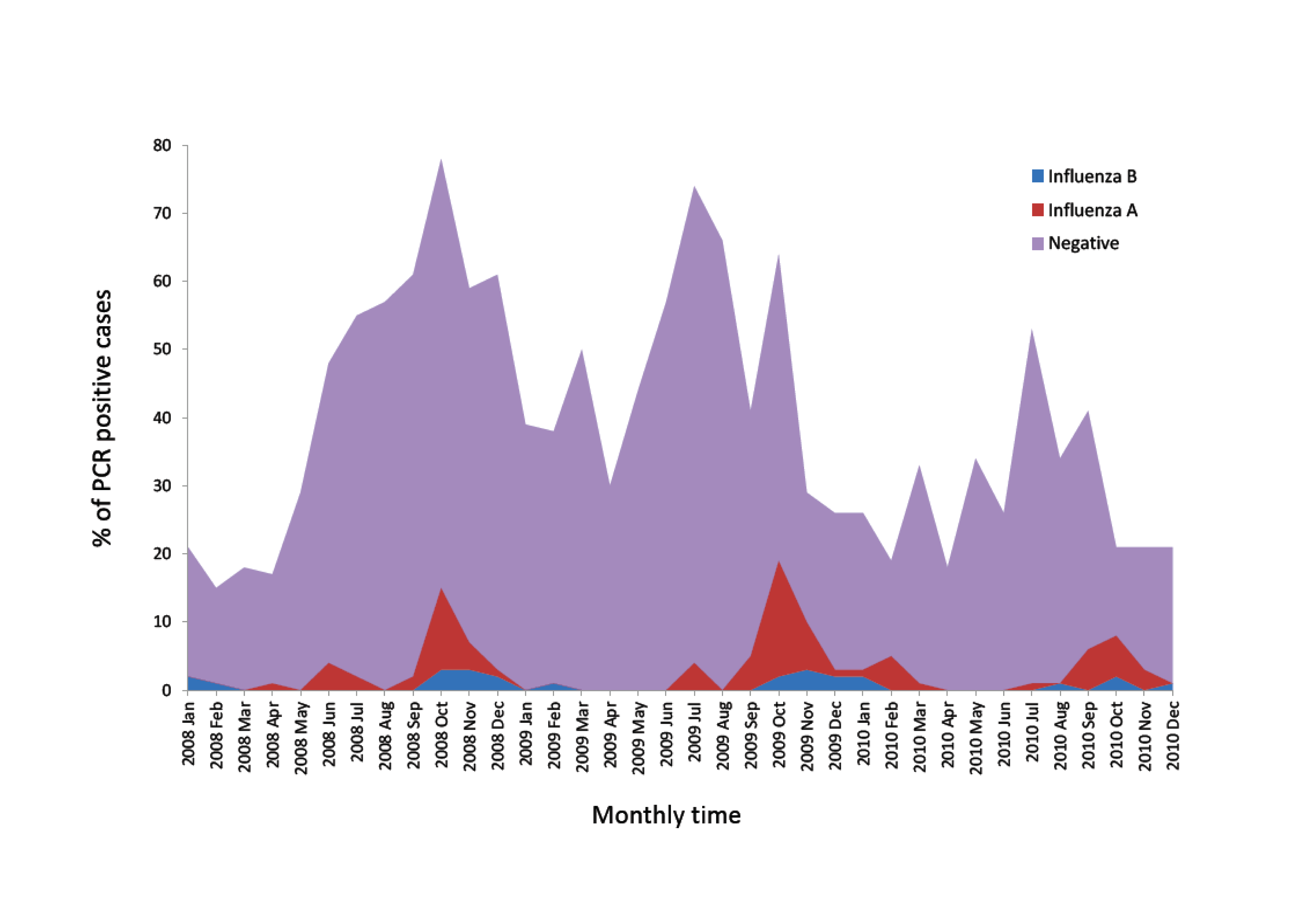

Supplement: Figure S4 — Temporal distribution of the influenza (A and B) cases observed in the study sites (January 2008–December 2010). (TIF) [file pone.0095868.s004.tif]
