# Supplementary material for: Acute Undifferentiated Febrile Illness in Rural Cambodia: A 3-Year Prospective Observational Study
Source: PLoS One. 2014 Apr 22;9(4):e95868. doi: 10.1371/journal.pone.0095868 (PMC3995936; doi:10.1371/journal.pone.0095868)
Supplement: Table S1 — Estimated detection thresholds of nucleic acid amplication tests (NAAT) used in the study. (DOCX) [file pone.0095868.s005.docx]

Table S1. Estimated detection thresholds of nucleic acid amplication tests (NAAT) used in the study.

| **Pathogen** | **Laboratory methods** | **Type of sample** | **Target (antigen or gene)** | **Estimated detection threshold** |
| --- | --- | --- | --- | --- |
| Malaria | PCR & Sequencing | DNA extracted from 200 µl red blood cells | *Plasmodium cytochrome b* gene | 0.08 copy/µl |
| Leptospirosis | PCR & Sequencing | DNA extracted from 200 µl red blood cells | *16srRNA* gene | 16 copies/µl |
| Rickettsial disease | PCR & Sequencing | DNA extracted from 200 µl red blood cells | *ompB* gene | 200 copies/µl |
|  | PCR & Sequencing | DNA extracted from 200 µl red blood cells | *gltA* gene | 200 copies/µl |
| Scrub Typhus | PCR & Sequencing | DNA extracted from 200 µl red blood cells | *47kDa* gene | 1000 copies/µl |
| Dengue | RT-PCR | RNA extracted from 200 µl of plasma | PrM/E gene - Dengue virus 1-4 | 100 copies/ml |
| Influenza | RT-PCR | RNA extracted from throatswab | *M* gene - Influenza A and B | 4 copies/µl (IA) and 36 copies/µl (IB) |
